# A machine learning-based predictive model for complication risks in vacuum-assisted breast biopsy

**DOI:** 10.3389/fsurg.2025.1641441

**Published:** 2025-09-08

**Authors:** Sun Pingdong, Shao Xinran, Shen Yunzhi, Sun Yihan, Zheng Shipeng, Li Yan, Li Qiushi, Zheng Jipeng, Ruan Ting, Wu Wenjun, Yao Shengsheng, Li Gang, Liu Jinrui, Ju Xingai, Fei Xiang, Cui Jianchun

**Affiliations:** ^1^ Department of Thyroid and Breast Surgery, People's Hospital of China Medical University, Shenyang, China; ^2^ Graduate School, Dalian Medical University, Dalian, China; ^3^ Liaoyang County Hospital, Liaoyang, China; ^4^ Department of Cardiology, People's Hospital of China Medical University, Shenyang, China; ^5^ Department of Breast Surgery, The First Affiliated Hospital of Zhengzhou University, Zhengzhou, China; ^6^ Department of Breast Surgery, The Second People’s Hospital of Hami, Hami, China; ^7^ Department of General Medicine, People's Hospital of China Medical University, Shenyang, China; ^8^ The Fourth Affiliated Hospital, Liaoning University of Traditional Chinese Medicine, Shenyang, China; ^9^ Changchun University of Chinese Medicine, Changchun, China; ^10^ Department of Emergency Medicine, People's Hospital of China Medical University, Shenyang, China

**Keywords:** vacuum-assisted breast biopsy, machine learning, prediction model, postoperative complications, breast tumor

## Abstract

**Background:**

Ultrasound-guided vacuum-assisted breast biopsy (VABB) has become the standard minimally invasive procedure for diagnosing and treating benign breast lesions. Despite its widespread adoption, postoperative complications such as bruising, residual tumors, and skin injury remain significant clinical challenges that can impact patient outcomes and satisfaction. Current risk assessment methods lack precision, highlighting the need for more sophisticated predictive tools.

**Methods:**

We conducted a multicenter retrospective study analyzing 1,064 VABB procedures performed at three medical centers between 2017 and 2025. Using a comprehensive set of 12 preoperative variables including tumor characteristics and anatomical relationships, we developed and validated six machine learning models. The random forest algorithm demonstrated superior performance in our five-fold cross-validation analysis, with particular strength in predicting postoperative bruising and operative duration.

**Results:**

Our predictive model achieved exceptional performance for bruising risk assessment (AUC 0.971, accuracy 96.7%) and moderate surgical duration prediction. SHAP analysis identified three key predictive features: tumor size (mean SHAP value 0.32), blood flow grade (0.28), and distance to pectoralis muscle (0.25). The model maintained strong performance in external validation (AUC 0.945), confirming its generalizability. However, prediction of rare complications like tumor residual showed limited effectiveness (AUC 0.68).

**Conclusions:**

This study presents a clinically validated machine learning tool that accurately predicts common VABB complications, particularly postoperative bruising. By incorporating specific anatomical and tumor characteristics into preoperative planning, surgeons can better anticipate and potentially mitigate these adverse outcomes. The model's integration into clinical practice could enhance surgical decision-making and improve patient counseling regarding expected recovery experiences.

**Clinical Trial Registration:**

https://www.chictr.org.cn/index.html, identifier ChiCTR2500095736.

## 1 Introduction

Benign breast tumors account for approximately 30 % of all breast diseases, with an increasing incidence in younger women (1, 2). Ultrasound-guided vacuum-assisted breast biopsy (VABB) is widely used for such tumors, and its indications have broadened owing to advances in imaging and surgical instruments (3). Nevertheless, postoperative cutaneous bruising, tumor residual, and skin damage have long been recognized as major complications that adversely affect patient prognosis (4). Operative difficulty is heightened and surgical efficacy diminished especially when breast tumors are excessively large or small, overly superficial or deep, unusually firm, situated adjacent to major vessels, or accompanied by marked pectoralis muscle. A complexity-grading and subtype-directed treatment paradigm for VABB was proposed by Cui et al. (2022), broadening procedural indications and helping to restrain overall complication rates; nevertheless, postoperative cutaneous bruising still occurs in up to 10% of conventional VABB cases and rises to about 20% in high-complexity procedures, underscoring the need for further optimization (5).

Machine learning (ML) is a type of artificial intelligence model that provides more accurate predictions without explicit programming; a variety of ML models are used to build information models based on big data that reveal potential relationships between multiple clinical predictors and outcomes (6–8), and analyze the underlying mechanisms of various complications (9), thus guiding effective protocols to reduce complications' occurrence.

This study aims to establish a ML model to explore the relationship between the aforementioned complex factors and complications of VABB, with the goal of improving prediction accuracy, optimizing surgical planning, enhancing surgical quality, and reducing the incidence of complications.

## 2 Materials and methods

### 2.1 Study cohort

Derived from 903 cases of VABB cases admitted to The People's Hospital of China Medical University between October 2017 and May 2024, which were randomly divided into the training set and test set in the ratio of 7:3; derived from 161 similar cases in the First Affiliated Hospital of Zhengzhou University and the Second People's Hospital of Hami City, Xinjiang, between March 2024 and January 2025, which served as a separate external validation set. All cases were graded according to the diagnostic criteria for above paradigm (5). Inclusion criteria: 1. Lesions classified as BI-RADS 4a or higher on ultrasound or BI-RADS 3 with a clear surgical indication; 2. patient preference for minimally invasive surgery; 3. Single or multiple lesions, provided that only one lesion required excision.Exclusion criteria: 1. those with bleeding tendency or who have recently taken anticoagulant drugs; 2. severe cardiopulmonary comorbidities precluding surgery; 3. Incomplete clinical or imaging data. This study was ethically reviewed by the Ethics Committee of Liaoning Provincial People's Hospital, Grant No. (2024) H058. All patients signed an informed consent form and agreed to use their data for this study. This study also passed the China Clinical Trial Registry, registration number ChiCTR2500095736.

According to the summary statistics in Table 1, the mean patient age was 41.98 ± 11.81 years, indicating that the cohort consisted largely of individuals in early-to-middle adulthood. Virtually all patients were non-lactating at the time of surgery, and lesions were distributed evenly between the left and right breasts. Almost half of the tumors were assigned Adler grade 0 for intralesional blood-flow signals; the proportion of patients declined progressively with increasing grades, suggesting that vascularity within and around most lesions was minimal. Calcifications were detected in only 7.0% of cases. Moderate breast ptosis was the most prevalent degree of sagging, observed in 50.4% of the study population.

**Table 1:** Descriptive statistics of relevant variables.

Clinical Characteristics and Statistical Results	
Characteristic	Statistical Results
Number of Patients	1,064
Age (Mean ± SD)	41.98 (11.81)
Breastfeeding Status (%)	
No	134 (12.6%)
Yes	930 (87.4%)
Left/Right Breast (%)	
Right	517 (48.6%)
Left	547 (51.4%)
Tumor Size (cm^2^) (Mean ± SD)	1.21 (2.02)
Blood Flow (Adler Classification) (%)	
0	501 (47.1%)
1	346 (32.5%)
2	176 (16.5%)
3	41 (3.9%)
Distance to Skin (cm) (Mean ± SD)	0.64 (0.25)
Distance to Pectoralis Muscle (cm) (Mean ± SD)	0.96 (0.59)
Distance to Nipple (cm) (Mean ± SD)	1.91 (1.04)
Distance to Gland Margin (cm) (Mean ± SD)	3.34 (1.06)
Tumor Direction (%)	
1 o'clock	78 (7.3%)
2 o'clock	101 (9.5%)
3 o'clock	113 (10.6%)
4 o'clock	64 (6.0%)
5 o'clock	46 (4.3%)
6 o'clock	34 (3.2%)
7 o'clock	25 (2.4%)
8 o'clock	61 (5.7%)
9 o'clock	153 (14.4%)
10 o'clock	190 (17.9%)
11 o'clock	79 (7.4%)
12 o'clock	119 (11.2%)
Calcification (%)	
No	990 (93.0%)
Yes	74 (7.0%)
Breast Ptosis (%)	
Normal	253 (23.8%)
Mild	213 (20.0%)
Moderate	536 (50.4%)
Severe	62 (5.8%)
Surgery Time (Short/Medium/Long) (%)	
Short	493 (46.3%)
Medium	557 (52.3%)
Long	14 (1.3%)
Bruising (%)	
No	840 (78.9%)
Yes	224 (21.1%)
Pathological Classification (%)	
Benign Lesions	1,029 (96.7%)
Inflammatory Lesions	14 (1.3%)
Malignant Lesions	21 (2.0%)
Skin Damage (%)	
No	1,023 (96.1%)
Yes	41 (3.9%)
Tumor Residual (%)	
No	1,054 (99.1%)
Yes	10 (0.9%)
Conversion to Surgery (%)	
No	1,040 (97.7%)
Yes	24 (2.3%)

Surgery time was predominantly short or medium, with prolonged procedures observed in only 1.3% of cases. Post-operative evaluation indicated that most patients developed no bruising, skin damage, or tumor residual. Pathological classification confirmed benign disease in 96.7% of tumors, and 97.7% of patients ultimately required no further conversion to surgery.

### 2.2 Surgical procedure

Experienced breast surgeons and radiologists (≥5 years' work experience) performed pre-operative ultrasound to localize lesions and record size, vascularity, and anatomical relationships. The needle entry site and trajectory were planned by the surgeon, and the target lesion was localized pre-operatively with a surface-projection plastic molds specifically designed for breast masses. The VABB was standardized into five maneuvers: adjust body position, buffer zone creation, lesion-groove positional adjustment, cavity water injection inspection, compression hemostasis. These above steps kept the cutting groove safely away from vessels, skin, pectoralis muscle, and nipple, while enabling real-time detection of minor bleeding and its precise control. The excised specimen was submitted for paraffin-embedded histopathological examination. After VABB, the resection cavity was packed with gauze at the skin surface and secured with a compressive elastic bandage. Forty-eight hours post-operatively, the bandage was removed, and a sports bra was applied to restrict breast motion, thereby minimizing pain and the risk of delayed hemorrhage.

### 2.3 Data preprocessing

This study's dataset consisted of 18 features, including 5 continuous and 13 categorical variables. Among them, 12 clinically meaningful features were selected as model inputs: age (years), breastfeeding status, laterality (left or right breast), tumor size (cm²), blood flow (Adler classification), distance from the tumor's upper margin to the skin (cm), distance from the lower margin to the pectoralis muscle (cm), distance from the proximal margin to the nipple (cm), distance from the distal margin to the glandular border (cm), tumor direction (clock-face positions), calcification (yes/no), and breast ptosis (classified by the degree of ptosis as normal, mild, moderate, or severe). In addition, six key outcome variables were included: surgery time (defined as the duration of ultrasound localization and vacuum-assisted excision, excluding anesthesia and compression time, and categorized as short <10 min, medium 10–20 min, or long >20 min), postoperative bruising (yes/no), pathological classification (benign, malignant, or inflammatory lesions), skin damage (yes/no), tumor residual detected by ultrasound at 3-month follow-up (yes/no), and conversion to surgery (yes/no). To align with ML requirements, tumor size was calculated as the product of its length and width to obtain the area, and surgery time and pathological classification were recoded as categorical variables according to the above classifications.

### 2.4 Model building

Multiclass classification models were developed within the mlr3 ML framework to obtain precise predictions for indices related to VABB (10). Six base learners were implemented: decision tree (recursive partitioning and regression trees; rpart) (11), random forest (ranger) (12), k-nearest neighbor (KNN) (13), extreme gradient boosting (XGBoost) (14), support vector machine (SVM) (15), and feed-forward neural network (nnet) (16).

During model training, a five-fold cross validation scheme was applied to each algorithm to maximize use of the limited dataset and to avoid overfitting.

### 2.5 Model evaluation

For model evaluation, accuracy and AUC are selected as the core evaluation metrics, and the optimal model is screened and extended to the test set and validation set for verification. In this study, SHapley additive interpretation (SHAP) values were used to assess the importance of overall features in the ML model with the best predictive performance (17).

### 2.6 Statistical analysis

Data were analyzed using the statistical package R (v4.1.3). Mean and standard deviation were used to describe continuous variables. Frequencies and percentages were used to describe categorical scalars. Statistical analysis was performed using the R packages table one, mlr3, mlr3benchmark, mlr3extralearner and shapviz. Statistical analysis were two-sided and *p*-values < 0.05 were considered statistically significant.

## 3 Results

### 3.1 Results of model building

**Figure 1:**
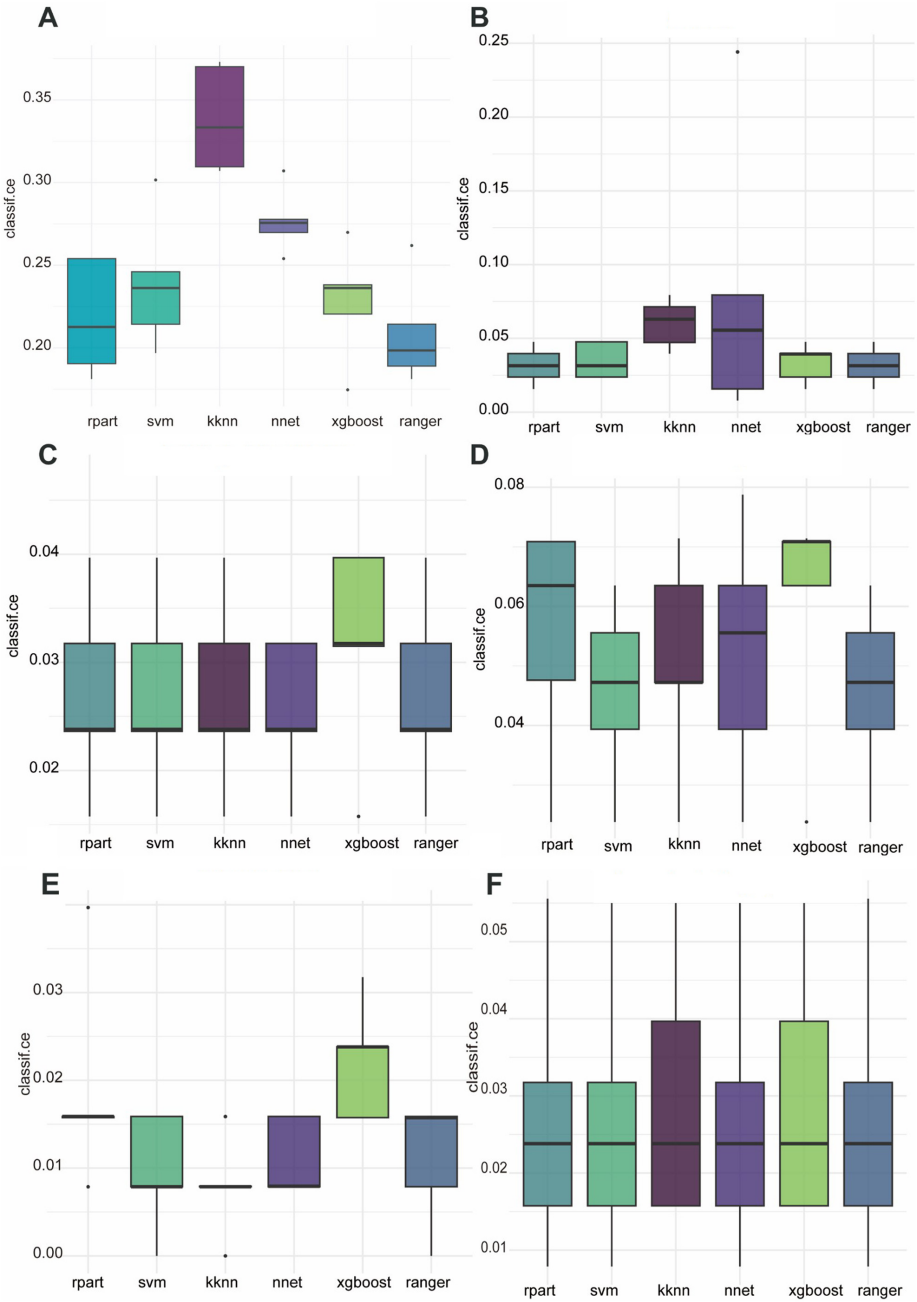
Classification error rates for six ML algorithms applied to the prediction of six postoperative complications. **(A)** Surgery time, **(B)** bruising, **(C)** pathological classification, **(D)** skin damage, **(E)** tumor residual, **(F)** conversion to surgery.

We first used six ML algorithms in the training set to construct a multiclassification model about the surgery time and a multiclassification model about the pathological classification, respectively. The classification error rates of different algorithms are shown in Figure 1. The ranger algorithm model about surgery time has the lowest error rate (0.021). The ranger algorithm for pathological classification had the same error rate as the rpart, NNet, and XGBoost, which were all 0.027.

In constructing a binary prediction model for postoperative complications, we compared the performance of six ML algorithms. The results show that the ranger algorithm performs best in predicting postoperative skin bruising with an error rate of 3.2%; the NNet is optimal in predicting postoperative skin damage with an error rate of 4.4%; the KNN has the lowest error rate in identifying tumor residual at 0.8%; and for the prediction to open surgery conversion, the SVM, the NNet, and the ranger collectively perform optimally, with an error rate of 1.1% each.

**Figure 2:**
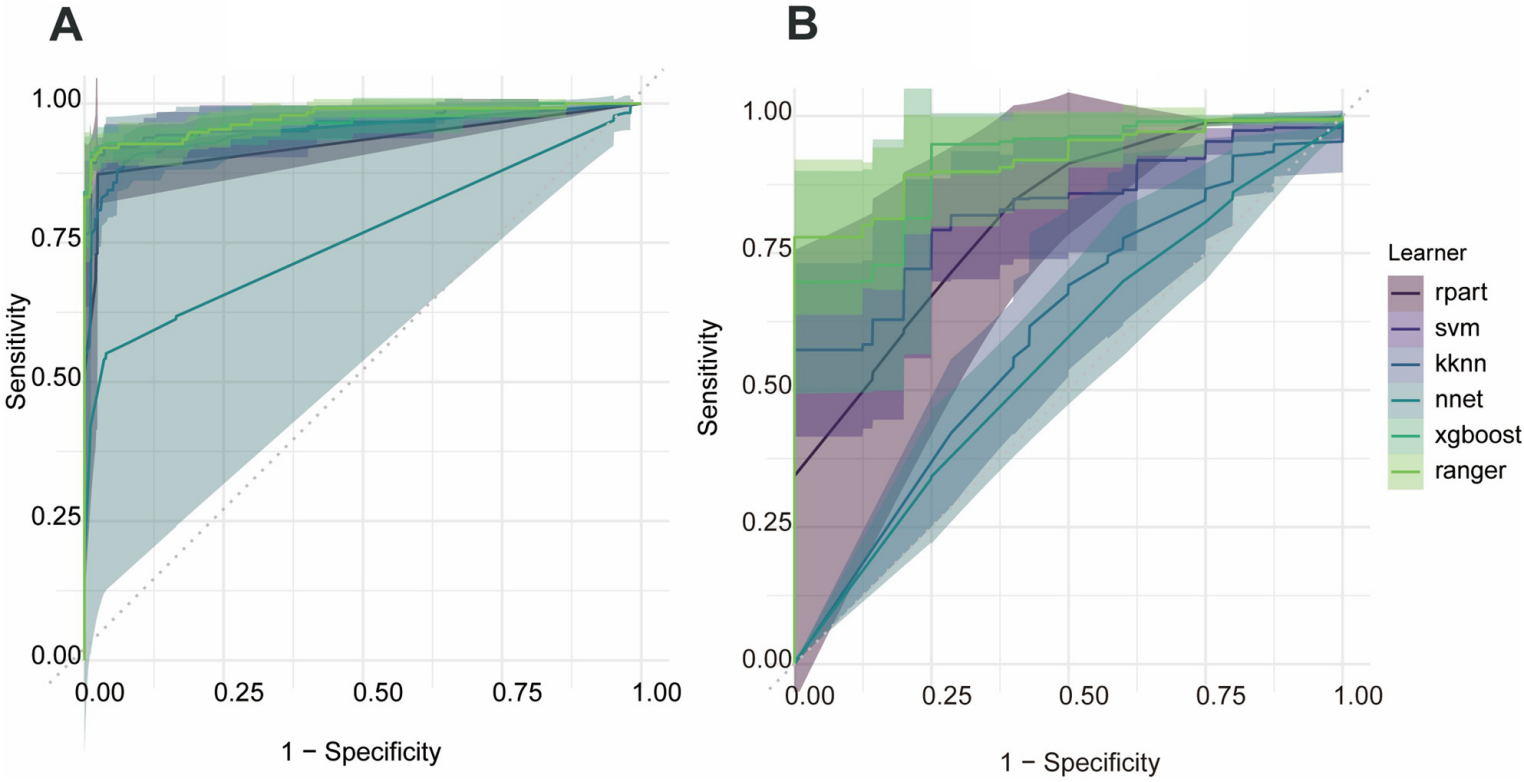
Receiver-operating-characteristic curves for the random-forest models predicting postoperative bruising and postoperative skin damage. **(A)** Bruising, **(B)** skin damage.

Receiver-operating-characteristic (ROC) analyses for all models are presented in Figure 2. The ranger algorithm yielded areas under the curve (AUCs) of 0.967 for bruising and 0.894 for skin damage. By contrast, the class distributions for conversion to surgery and tumor residual were extremely imbalanced, rendering the associated ROC curves unstable and the resulting AUC values of little significance.

### 3.2 Results of model validation

Based on the 5-fold cross-validation of the training set, the optimal results are selected for the operation. The model selection and the accuracy and multiclassification AUC on the test set and training set are shown in Supplementary Material S1.

**Figure 3:**
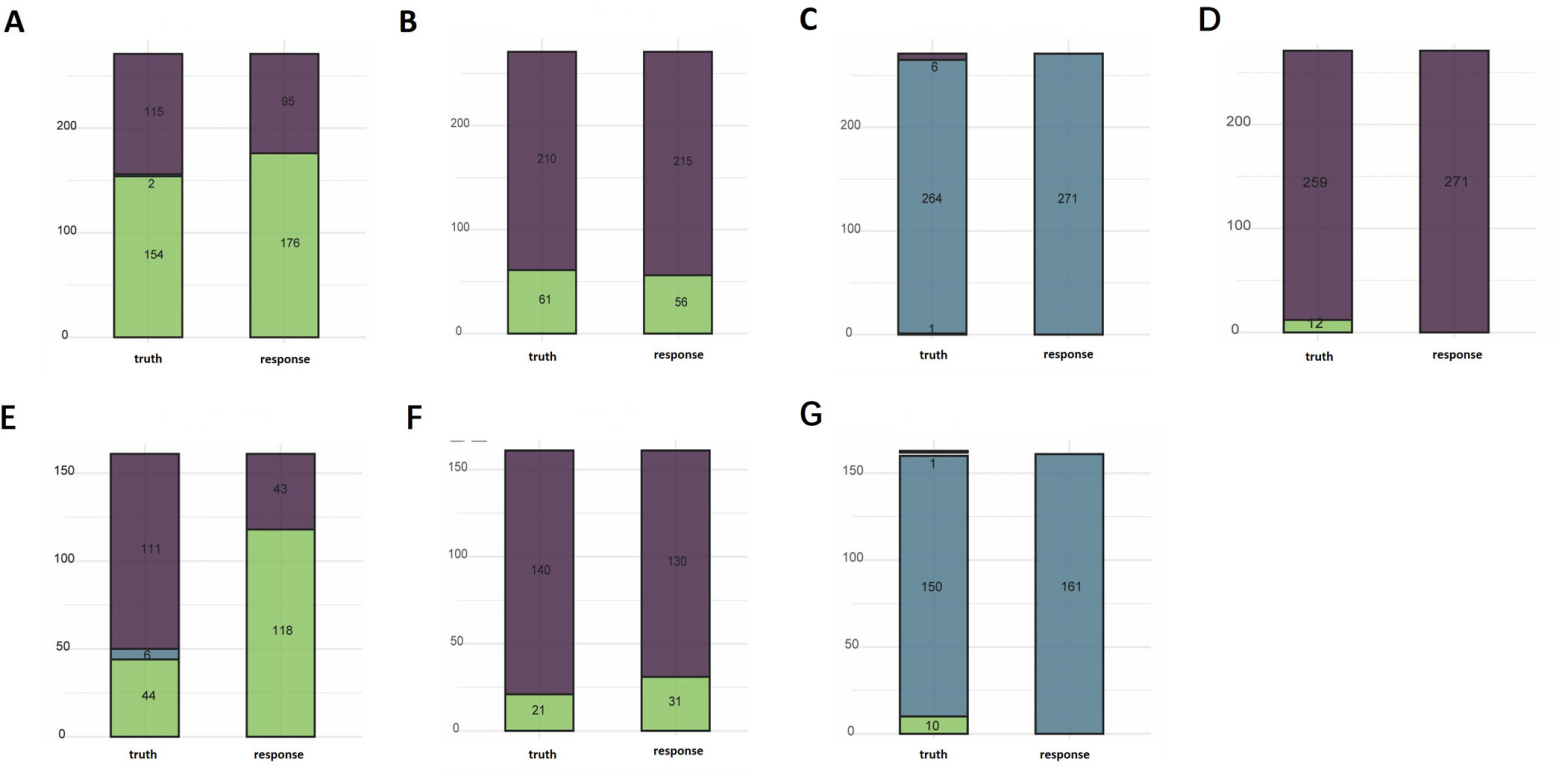
Performance profiles of the random-forest models for operative duration, postoperative ecchymosis/bruising, histopathology, and postoperative skin damage. **(A)** Surgery time-text, **(B)** bruising-text, **(C)** pathological-text, **(D)** skin damage-text, **(E)** surgery time-val, **(F)** pathological classification-val.

Regarding the multiclassification model for surgery time, the ranger algorithm gave the best results, with an accuracy of 0.797 and a multiclassification AUC of 0.818 on the test set, and an accuracy of 0.447 and a multiclassification AUC of 0.818 on the validation set. The classification model gave better results for short- and medium-length surgeries, but was not effective for long-length surgeries (Figure 3).

We used the ranger algorithm to construct a classification model and validate it on the full training set of postoperative bruises. The results showed an accuracy of 0.967 with an AUC of 0.971 on the test set. on the validation set the accuracy was 0.913 with an AUC of 0.945. The classification model showed good performance on the test set as well as on the external validation set (Figure 3).

Regarding the multi-classification model for pathological classification, three algorithms worked nearly the same in the 5-fold cross-validation. The ranger model was still chosen as the final model since the first few classification models were the most effective. The results showed an accuracy of 0.974 on the test set and 0.931 on the validation set. However, the model classified all samples as benign lesions and did not recognize the other two classifications that were less numerous (Figure 3).

Regarding the model of skin damage, the random forest algorithm had the best accuracy and AUC results. We used the ranger algorithm to construct a classification model for the full training set, and the results showed an accuracy of 0.956 on the test set. The validation set could not be validated because the skin damages in the validation set were all null (Figure 3).

### 3.3 Results of model interpretability

**Figure 4:**
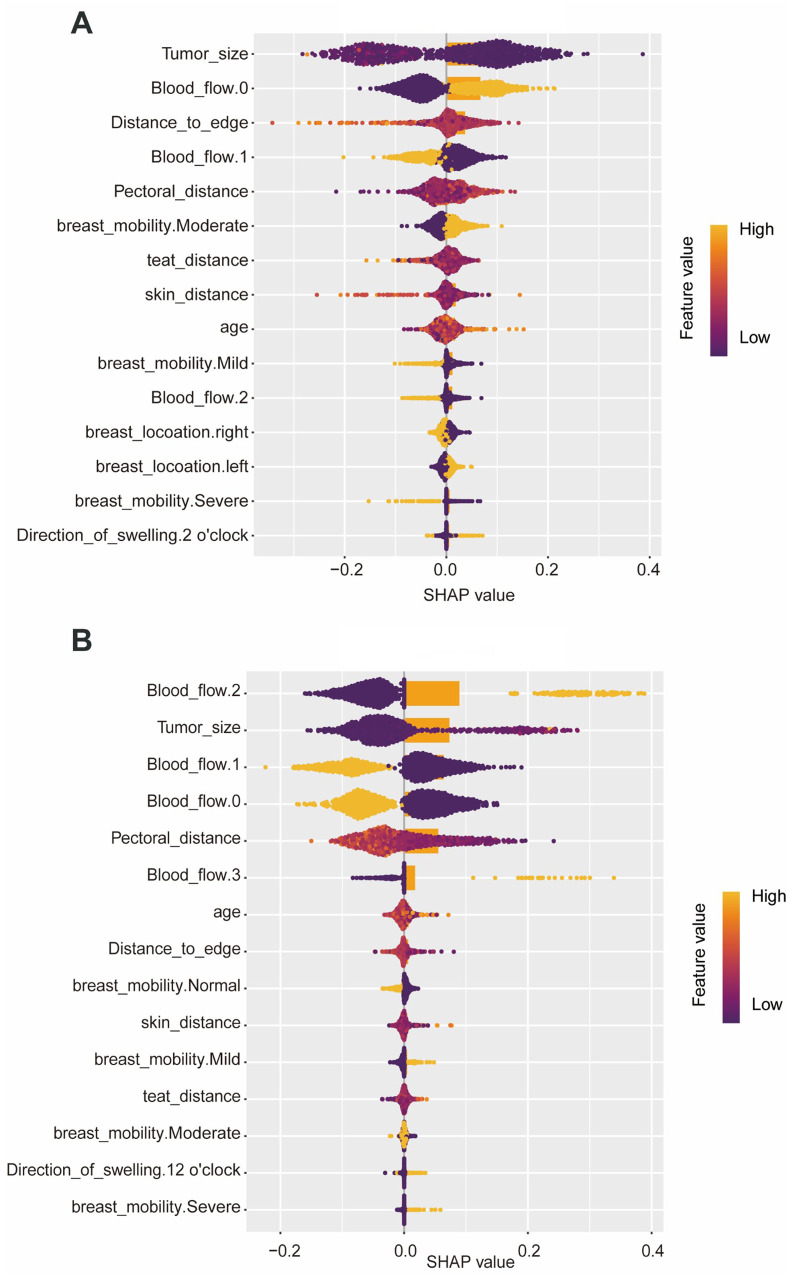
SHAP plots of each feature in the machine learning model for predicting surgery time and bruising. **(A)** Surgery time, **(B)** bruising.

The SHAP plot (Figure 4) shows the importance of each feature in the ML model for predicting the surgery time, bruising. The SHAP values show that the tumor size, blood flow, distance to gland margin, and distance to pectoralis muscle are important factors affecting the surgery time. In contrast, the likelihood of bruising was primarily determined by lesion size, blood-flow grade, and the distance to pectoralis muscle**.**

## 4 Discussion

Ultrasound-guided VABB is now a well-established technique. The 2017 VABB guidelines stipulated that excision should be restricted to lesions ≤3 cm in maximal diameter (18). In the 2021 revision, however, it was emphasized that the probability of residual disease rises in direct proportion to tumor size once the diameter exceeds 2 cm, and no explicit upper size limit was imposed (19). The 2017 document further classified lesions situated near the nipple–areolar complex or adjacent to breast implants as relative contraindications (18), whereas the 2021 guidelines recommended VABB as the preferred approach for tumors <1 cm, particularly when located close to the chest wall or an implant (19). The progressive relaxation of surgical indications has been accompanied by a tangible rise in procedural difficulty and complexity. Among the 1,064 cases analyzed, the prevalence of individual complicating factors was as follows: excessive lesion size, 14%; very small lesions, 39%; excessive depth, 67%; excessive superficiality, 95%; close proximity to major vessels or marked hyper-vascularity, 20%; calcification, 6%; and excessive Breast Ptosis, 56%. When cases were stratified by cumulative complexity, mild complexity (one–two factors) accounted for 30% of procedures and was associated with a 7% complication rate; moderate complexity (three–four factors) comprised 57% of procedures with a 25% complication rate; and severe complexity (five or more factors) represented 8.4% of procedures but carried an 86% complication rate. These data demonstrate a strong positive association between procedural complexity and the incidence of postoperative complications in vacuum-assisted breast excision.

By elucidating the correlation between procedural complexity and postoperative complications in VABB, prophylactic, case-specific measures can be implemented pre-operatively in high-risk patients, thereby enhancing surgical quality and safeguarding clinical outcomes.

Surgery time and bruising were identified as outcome variables with unequivocally positive predictive signals. For both endpoints, the ranger algorithm demonstrated the lowest misclassification rate and achieved the highest precision and AUC values in the test set. SHAP analysis further revealed that lesion size, intralesional blood-flow grade, and the distance to gland margin were the principal determinants of surgery time and bruising. Surgery time and bruising were identified as endpoints with strong positive predictive signals. Tumor size, intralesional vascularity, and the distances to gland margin, pectoralis muscle, and skin proved to be the principal determinants of surgery time, whereas tumor size, vascularity, and the distance to pectoralis muscle exerted the greatest influence on bruising. Excessive tumor size, overly superficial or deep location, and close proximity to major vessels or marked hyper-vascularity constituted the complexity factors most strongly associated with prolonged operative time and with the development of bruising. Careful intra-operative attention to these parameters should therefore help prevent unnecessarily lengthy procedures and reduce the incidence of postoperative cutaneous ecchymosis and hematoma.

Across the full cohort of 1,064 cases, procedures of short (<10 min) and moderate (10–20 min) surgery time accounted for 46.3% and 52.3%, respectively—i.e., 98.6% were completed within 20 min, fully consistent with the minimally invasive and rapid nature of VABB. Although prolonged operations (>20 min) were uncommon (*n* = 14; 1.3%), the longest procedure extended to 60 min.

Among these cases, the incidence of bruising reached 21.1%, while tumor residual, skin damage, and conversion to surgery occurred in 0.9%, 3.9%, and 2.3%, respectively—together representing over 50% of all such rare complications in the series. Notably, these operations typically involved four to six complicating factors, underscoring that meticulous management of moderate-to-severe complexity cases is pivotal for reducing the overall rate of infrequent but clinically significant complications.

To optimize the safety and technical quality of minimally invasive VABB, each complicating factor was classified and paired with a targeted mitigation strategy. Type I complexity—tumors that are excessively large or small—requires precise pre-operative localization. Type II complexity refers to lesions situated close to the skin, pectoralis muscle, or major vessels; in these cases, injection of a perilesional buffer zone and adjustment of patient positioning are recommended to widen the interval between the tumor and adjacent critical structures. Type III complexity is characterized by marked breast ptosis and is best managed through enhanced fixation to prevent displacement of either the tumor or the cutting groove. A special subtype encompasses heavily calcified lesions; these are most safely addressed by coring the tissue circumferentially to free the mass, followed by retrieval with curved forceps (5, 20). Finally, comprehensive, workflow-based management of the entire excision procedure has been advocated to further improve surgical quality and patient safety (21).

Several limitations were encountered while developing the complication-risk prediction model for VABB. The overall sample size—especially the number of positive events for prolonged surgery time, skin damage, and tumor residual—was limited. This resulted in a severe class imbalance, particularly for rare events such as tumor residual and surgical conversion, causing the model to exhibit a bias toward the majority classes. Consequently, although the ranger model achieved high aggregate accuracy in both the test and validation sets, its ability to discriminate minority classes was constrained. A more balanced and larger dataset, or effective class-balancing strategies, such as SMOTE, could potentially improve the model's ability to detect these infrequent but critical outcomes. Future investigations should therefore refine the model architecture, expand the dataset, or implement more effective class-balancing strategies to improve performance for these infrequent outcomes. At the technical level, it is recommended that synthetic-minority oversampling methods such as SMOTE be adopted to improve class balance, and that multicenter collaboration be pursued to enlarge both the size and diversity of the dataset. Although the models displayed satisfactory predictive performance, the underlying decision rationale remains insufficiently transparent; future work should therefore incorporate interpretability techniques—e.g., SHAP value analysis—to enhance clinical trust. For routine clinical deployment, operative variables such as surgeon experience, device selection, and long-term follow-up outcomes should be integrated, and a real-time prediction module compatible with surgical-navigation systems should be developed. The absence of long-term follow-up data in this study limits the understanding of the model's potential in predicting postoperative complications and patient outcomes over time. The present study was limited to VABB, and the generalizability of the models to other breast procedures has yet to be established. To address this, the research paradigm will be expanded to encompass additional breast procedures—including radical mastectomy and breast-conserving surgery—and model generalizability will be evaluated through transfer-learning approaches. Ultimately, deep interdisciplinary collaboration between clinical medicine and biomedical informatics will facilitate the transformation of these predictive models into precise, intelligent decision-support systems for routine clinical use.

## 5 Conclusion

In summary, the VABB prediction model developed in this study demonstrated substantial clinical value—most notably in forecasting surgery time and bruising—and can assist surgeons in formulating more precise operative strategies. Further refinements should focus on enhancing class balance within the dataset to strengthen the model's overall practicality and reliability.

## Data Availability

The original contributions presented in the study are included in the article/Supplementary Material, further inquiries can be directed to the corresponding author/s.
